# Gut microbiota regulates hepatic ischemia–reperfusion injury‐induced cognitive dysfunction via the HDAC2‐ACSS2 axis in mice

**DOI:** 10.1111/cns.14610

**Published:** 2024-02-09

**Authors:** Yanbo Liu, Zhen Li, Tianning Sun, Zhixiao Li, Anne Manyande, Hongbing Xiang, Zhigang He

**Affiliations:** ^1^ Department of Anesthesiology and Pain Medicine, Hubei Key Laboratory of Geriatric Anesthesia and Perioperative Brain Health, Wuhan Clinical Research Center for Geriatric Anesthesia, Tongji Hospital, Tongji Medical College Huazhong University of Science and Technology Wuhan China; ^2^ School of Human and Social Sciences University of West London London UK; ^3^ Department of Emergency Medicine, Tongji Hospital, Tongji Medical College Huazhong University of Science and Technology Wuhan China; ^4^ Department of Critical Care Medicine, Tongji Hospital, Tongji Medical College Huazhong University of Science and Technology Wuhan China

**Keywords:** cognitive dysfunction, gut microbiota, HDAC2‐ACSS2 axis, hepatic ischemia, reperfusion injury

## Abstract

**Aims:**

Hepatic ischemia–reperfusion injury (HIRI) resulting from hepatic inflow occlusion, which is a common procedure in liver surgery is inevitable. Previous research has confirmed that the cognitive dysfunction induced by HIRI is closely related to dysbiosis of the gut microbiota. This research aims to investigate the mechanisms underlying this complication.

**Methods:**

C57BL/6 mice underwent hepatic ischemia experimentally through the occlusion of the left hepatic artery and portal vein. To assess the HDAC2‐ACSS2 axis, gut microbiota transplantation. Enzyme‐linked immunosorbent assay and LC/MS short‐chain fatty acid detection were utilized.

**Results:**

The findings indicated a notable decline in ACSS2 expression in the hippocampus of mice experiencing hepatic ischemia–reperfusion injury, emphasizing the compromised acetate metabolism in this particular area. Furthermore, the cognitive impairment phenotype and the dysregulation of the HDAC2‐ACSS2 axis could also be transmitted to germ‐free mice via fecal microbial transplantation. Enzyme‐linked immunosorbent assay revealed reduced Acetyl‐coenzyme A (acetyl‐CoA) and Acetylated lysine levels in the hippocampus.

**Conclusion:**

These findings suggest that acetate metabolism is impaired in the hippocampus of HIRI‐induced cognitive impairment mice and related to dysbiosis, leading to compromised histone acetylation.

## Graphical abstract

The dysregulation of the HDAC2‐ACSS2 axis and abnormal SCFA metabolism in the hippocampus of HIRI mice could be transferred to germ‐free mice through the gut microbiota.

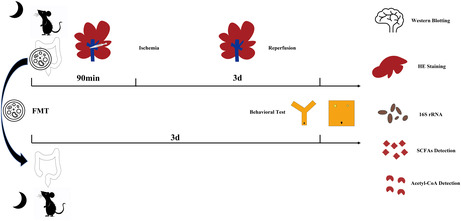

## 1 INTRODUCTION

Hepatic inflow occlusion is a common procedure in liver surgery.^1^, ^2^, ^3^ Studies have shown that occluding the hepatic portal can significantly reduce intraoperative bleeding and improve surgical visualization.^4^, ^5^, ^6^ However, hepatic ischemia–reperfusion injury (HIRI), an inevitable consequence of such therapy, may have adverse effects on patient prognosis, contributing to detrimental effects such as early liver failure, tissue damage, and even liver transplant failure.^7^, ^8^, ^9^, ^10^

Prior research has established that an imbalance in the gut microbiota is implicated in the progression of diverse conditions, including Crohn's disease, diabetes, and hypertension.^11^, ^12^, ^13^ The gut microbiome and the connection between the gut and the brain have gained significant attention in recent years in numerous studies investigating the pathophysiology of different diseases.^14^, ^15^, ^16^, ^17^ Multiple pathways, including inflammation in the central nervous system, alteration of microbial signals, and changes in intestinal barrier permeability, have been identified as potential causes of cognitive impairment due to dysbiosis of the gut microbiota.^16^, ^18^, ^19^

As is well known, histones are one of the fundamental components of DNA packaging.^20^ Histone post‐translational modifications, which are a significant component of epigenetics, have been demonstrated to have a vital impact on life regulation in recent times.^21^, ^22^ Histone post‐translational modifications include methylation, phosphorylation, ubiquitination, etc.^23^, ^24^ Cognitive function has been found to have a strong connection with histone acetylation/deacetylation, as demonstrated by previous studies.^25^, ^26^ Histone deacetylase 2 (HDAC2) and acetyl‐CoA synthetase 2 (ACSS2) are key enzymes that regulate this balance. ACSS2 uses acetate as a substrate to synthesize Acetyl‐CoA, directly promoting histone acetylation, which has an impact on learning and memory.^27^, ^28^ Previous studies have reported that mice with reduced ACSS2 expression exhibit impaired cognitive function, possibly related to the mechanism mentioned below.^29^

Previous research, including our team's previous work, has confirmed that HIRI can induce cognitive dysfunction in mice.^30^, ^31^, ^32^ The occurrence of this cognitive impairment is closely related to gut microbiota and hippocampal lipid metabolism. To further explore the mechanisms underlying hepatic ischemia–reperfusion‐induced cognitive dysfunction, our research group employed techniques including gut microbiota transplantation and LC/MS short‐chain fatty acid detection to investigate changes in the hippocampus of mice subjected to HIRI. This provides a preliminary theoretical basis for clinical practices aimed at treating cognitive dysfunction induced by HIRI.

## 2 MATERIALS AND METHODS

### 2.1 Animals

The 6–8 weeks aged male SPF C57BL/6 mice were acquired from Tongji Hospital's Animal Centre. They were kept in a colony room with controlled temperature and a standard 12‐h light control cycle (8:00, lights on; 20:00, lights off). The HIRI surgeries were performed at Zeitgeber Time 12 (ZT12, 20:00).

### 2.2 The experimental model of HIRI in mice

All procedures were performed during nighttime. As previously stated in earlier research,^31^, ^32^ mice were anesthetized using pentobarbital sodium solution (50 mg/kg, i.p). Subsequently, the rodents were positioned on an animal operating table and a median incision was performed to reveal the liver region following disinfection of the abdominal skin. The hepatic ischemia–reperfusion injury group (HIRI group) was subjected to previously described procedures. In short, ischemia was caused by blocking the left hepatic artery and portal vein for a duration of 90 min with the use of an artery clamp. The control group (Control group) experienced dissection of the hepatic artery and hepatic vein without any blockage. Throughout the surgery, the mice were maintained at a comfortable temperature, and lidocaine was applied to the surgical incision post‐procedure. Experiments and analysis were carried out 72 h after reperfusion, where behavioral tests were performed and samples of feces, liver tissue, serum, and hippocampal tissue were collected.

### 2.3 Behavioral test

Behavioral experiments were carried out on both the HIRI and control groups 72 h after reperfusion. For the F‐HIRI and F‐Sham group, experiments to observe behavior were performed on the day after the last time of microbiota transfer. To examine movement, fear, and repetitive actions, the open field experiment was employed. The Y‐maze test was performed to evaluate spatial working and reference memory, while non‐spatial visual learning memory was assessed through the novel object recognize test. The test apparatus was cleaned with medicinal alcohol after each experiment to reduce the influence of odor. Intelligent video tracking software automatically captures and examines behavioral data.

### 2.4 Open Field Test (OFT)

As described previously,^23^, ^24^, ^25^ after habituation for 3 h, the mice were placed into the center of an opaque open‐field chamber (40 cm in length, 40 cm in width, and 40 cm in height). Rodents were allowed to move freely under dim light conditions (300 lux) for 5 min, and the total travel distance and time spent in the center area were analyzed.

### 2.5 Novel Object Recognize Test (NORT)

The NORT test was executed following the previous description.^33^, ^34^ As previously mentioned, two indistinguishable items were positioned at two sides, 6 cm away from each edge, as explained earlier.^25^ During the training phase, the animal had the opportunity to freely investigate its surroundings for a duration of 5 min following the adjustment period, excluding any presence of objects. The duration of exploration around each object was meticulously documented. Following a 2‐h interval, the mice were reintroduced to the identical apparatus and given 5 min to freely investigate, during which one of the indistinguishable objects was substituted with a new object (test session). The time spent exploring the novel (NT) and familiar objects (FT) was measured and utilized for computing the recognition index.

### 2.6 Y‐Maze test

As described earlier,^35^ the experiment took place in a Y‐shaped enclosure consisting of three separated arms, each positioned at a 120° angle. The dimensions of the enclosure were 30 cm in length, 8 cm in width, and 15 cm in height. The initial limb (animal entrance) and the typical limb were consistently left unclosed, while the additional limb was obstructed during the initial phase. Afterward, the mice investigated the pair of accessible arms for a duration of 5 min. Following a duration of 2 h, all three arms were unlocked, granting the mice unrestricted entry to the three arms for a period of 5 min (test trial). For analysis, we documented the duration spent in each arm and the count of mice entering the new arm.

### 2.7 Pseudo germ‐free mice and fecal microbial transplantation

The depletion of gut microbiome and fecal microbial transplantation (FMT) were conducted following earlier established protocols.^36^ Mice were given vancomycin (100 mg/kg), neomycin sulfate (200 mg/kg), metronidazole (200 mg/kg), and ampicillin (200 mg/kg) through gavage once a day for 4 days in order to eliminate their intestinal bacteria. To perform fecal microbial transplantation, fecal matter from donor mice in the Sham and HIRI groups was gathered separately and mixed with phosphate‐buffered saline (PBS) until it reached a concentration of 0.125 g/mL. This suspension was orally administered to mice through gavage (0.15 mL) daily for consecutive 3 days. Pseudo germ‐free mice receiving FMT were divided into two groups: F‐Sham and F‐HIRI based on the suspension they received. Behavioral tests were performed on the following day after FMT. Full details are given in Figure S1.

### 2.8 16S rRNA microbiome sequencing

Following the established protocols,^29^ fecal samples were gathered, promptly frozen, and stored at −80°C subsequent to the conclusion of behavioral assessments. The sequencing of the microbiota's 16S rRNA was performed at OE Biotech Co., Ltd., Shanghai, China. Raw data in FASTQ format was generated by performing DNA extraction, amplification, library construction, and sequencing on the fecal samples using an Illumina Inc. platform (San Diego, CA, USA) and the services offered by OE Biotech Company (Shanghai, China). The software and platform used for additional bioinformatic analysis of the raw data were provided by OE Biotech Co., Ltd. based in Shanghai, China.

### 2.9 Western blotting

Following deep anesthesia induced by sodium pentobarbital, the mice were sacrificed, and the hippocampus were obtained and pulverized in a lysis buffer. After centrifugation, the protein concentration of the liquid above was measured using the bicinchoninic acid kit provided by Boster, Ltd., Wuhan, China. Afterward, 20 μg of every nuclear protein sample underwent separation on 10% SDS‐PAGE gels and were then transferred onto a polyvinylidene fluoride membrane. Afterward, the membrane was obstructed using 5% BSA for a duration of 1 h at room temperature. Following this, it was incubated with specific primary antibodies overnight at a temperature of 4°C. Following three washes, the blots were exposed to the suitable secondary antibody for a duration of 1 h at ambient temperature. Immuno‐reactivity was quantified by densitometry using enhanced chemiluminescence.

The antibodies utilized in this study included anti‐GAPDH (A19056; ABclonal) at a dilution of 1:1000, anti‐ACSS2 (A12334; ABclonal) at a dilution of 1:1000, and anti‐HDAC2 (A19626; ABclonal) at a dilution of 1:1000.

### 2.10 Enzyme‐linked immunosorbent assay

The levels of Acetyl‐CoA and acetylated lysine in the hippocampus of mice were quantified using the corresponding ELISA kit (Bio‐Swamp Co., Ltd.). The complete process was carried out in accordance with the guidelines provided by the manufacturer.

### 2.11 Determination of Short‐chain fatty acids (SCFAs)

In brief, 30 mg samples were subjected to crushing and homogenization, followed by treatment with methanol and L‐2‐chlorophenylalanine. The ACQUITY UPLC I‐Class system (Waters Corporation, Milford, CT, USA) and VION IMS QTOF Mass spectrometer (Waters Corporation, Milford, CT, USA) were utilized to conduct metabolic profiling in both electrospray ionization (ESI) positive and ESI negative ion modes. Progenesis QI V2.3 software (Non‐linear Dynamics, Newcastle, UK) was utilized to process the initial LC–MS data. This involved baseline filtering, peak identification, integration, retention time correction, peak alignment, and normalization. Afterwards, R was used to perform principal component analysis and orthogonal partial least‐squares‐discriminant analysis in order to investigate the relationship between the two groups.

### 2.12 Statistical analysis

All quantification data were expressed as means ± SEM, and error bars represented SEM. The evaluation of the normality distribution was conducted by utilizing the Kolmogorov–Smirnov test, while distinctions between groups were determined through an unpaired *t*‐test. Significant differences were determined when the confidence limits surpassed 95% (*p* < 0.05).GraphPad Prism 6.0 and Adobe Photoshop 22.1.1 were utilized for statistical analyses and creating graphs, excluding certain data obtained from 16S rRNA microbiome sequencing analyses and LC–MS.

## 3 RESULTS

### 3.1 HIRI‐induced cognitive deficits and dysbiosis of gut microbiota in mice

**FIGURE 1:**
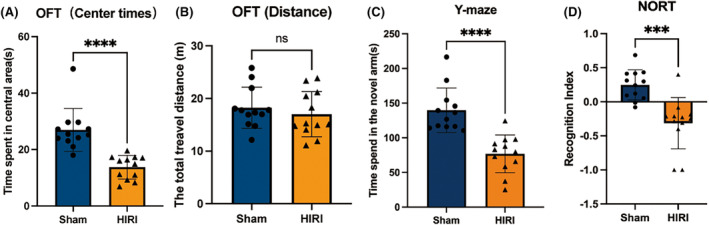
HIRI‐induced cognitive deficits in mice under ZT12. (A) Time spent in the central area of OFT. (B) Total travel distance of open field test (OFT). (C) Time spent in the novel arm of the Y‐maze test. (D) Recognition index of novel object recognition test (NORT). *N* = 12 for the Sham and HIRI group. Comparisons between groups were made with an unpaired *t*‐test. Data are presented as the mean ± SEM. ***p < 0.001, ****p < 0.0001; ns, not significant.

**FIGURE 2:**
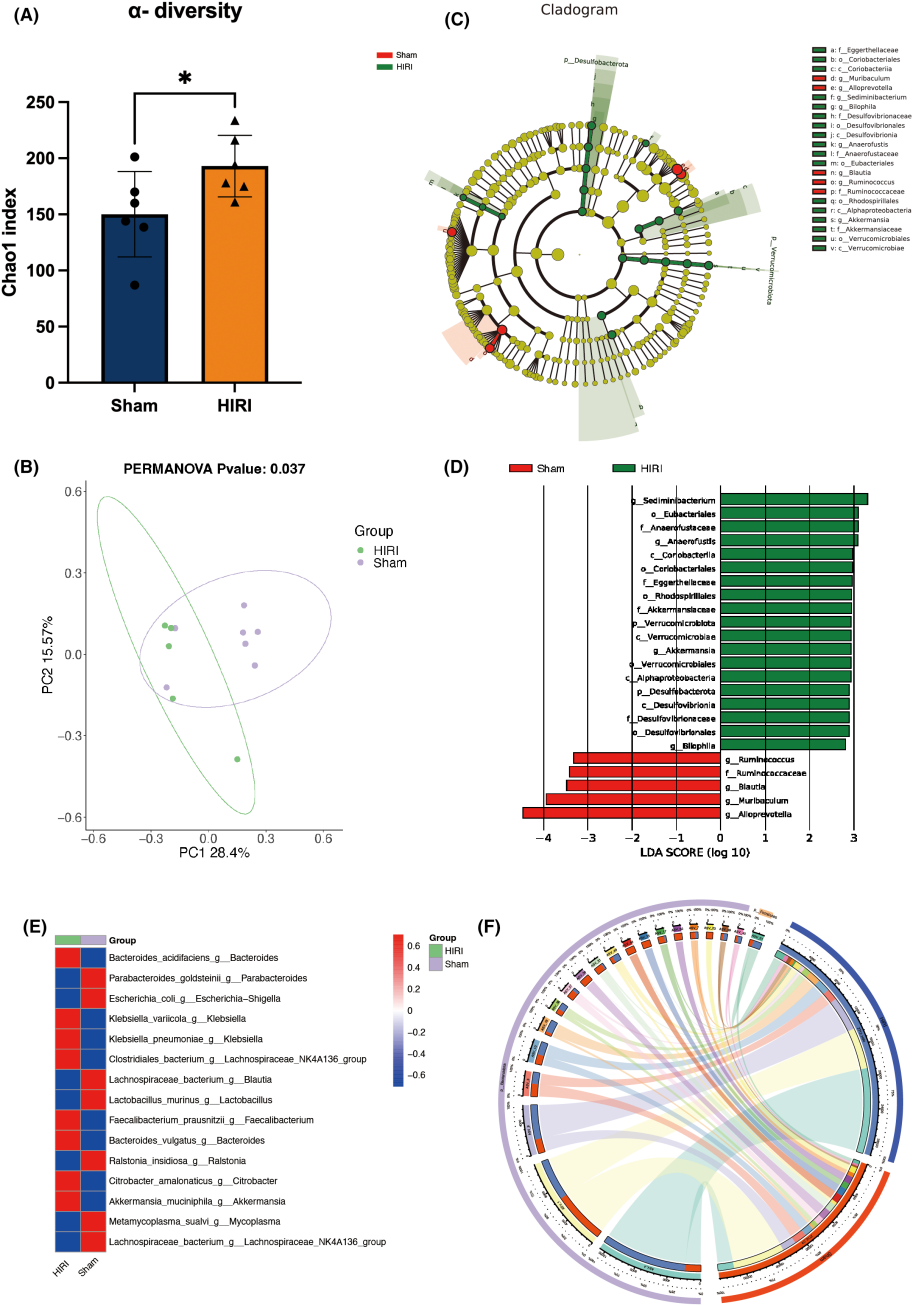
HIRI procedure induced gut microbiota alternation in mice compared with Sham group. (A) α‐diversity Chao1 index. (B) Principal Co‐Ordinates Analysis (PCoA). (C and D) Linear discriminant analysis Effect Size (LEfSe) analysis. (E) Top 15 different species between the two groups. (F) Circos plot. *N* = 6 for the Sham and HIRI group. Comparisons between groups were made with an unpaired *t*‐test. Data are presented as the mean ± SEM. *p < 0.05.

After a 3‐day reperfusion period, both groups of mice underwent cognitive function tests (Figure 1). In line with prior research results,^31^, ^32^ mice that experienced hepatic ischemia at night (HIRI group) displayed notable cognitive impairment in comparison to the Sham group, clearly demonstrating the surgery's substantial effect on the central nervous system. The representative images of behavior tests of Sham and HIRI were provided in Figure S3. We further performed 16S rRNA analysis on the fecal samples of the two groups (Figure 2). The findings revealed that there were 63 distinct bacteria in the fecal samples that exhibited variation between the two groups across all levels. This implies a significant disparity in the makeup of gut microbiome between the two groups, specifically in terms of modified quantities of *Blautia*, *Akkermansia*, and *Anaerofustis*.

### 3.2 HIRI led to dysregulation of the HDAC2‐ACSS2 axis in the hippocampus of mice

**FIGURE 3:**
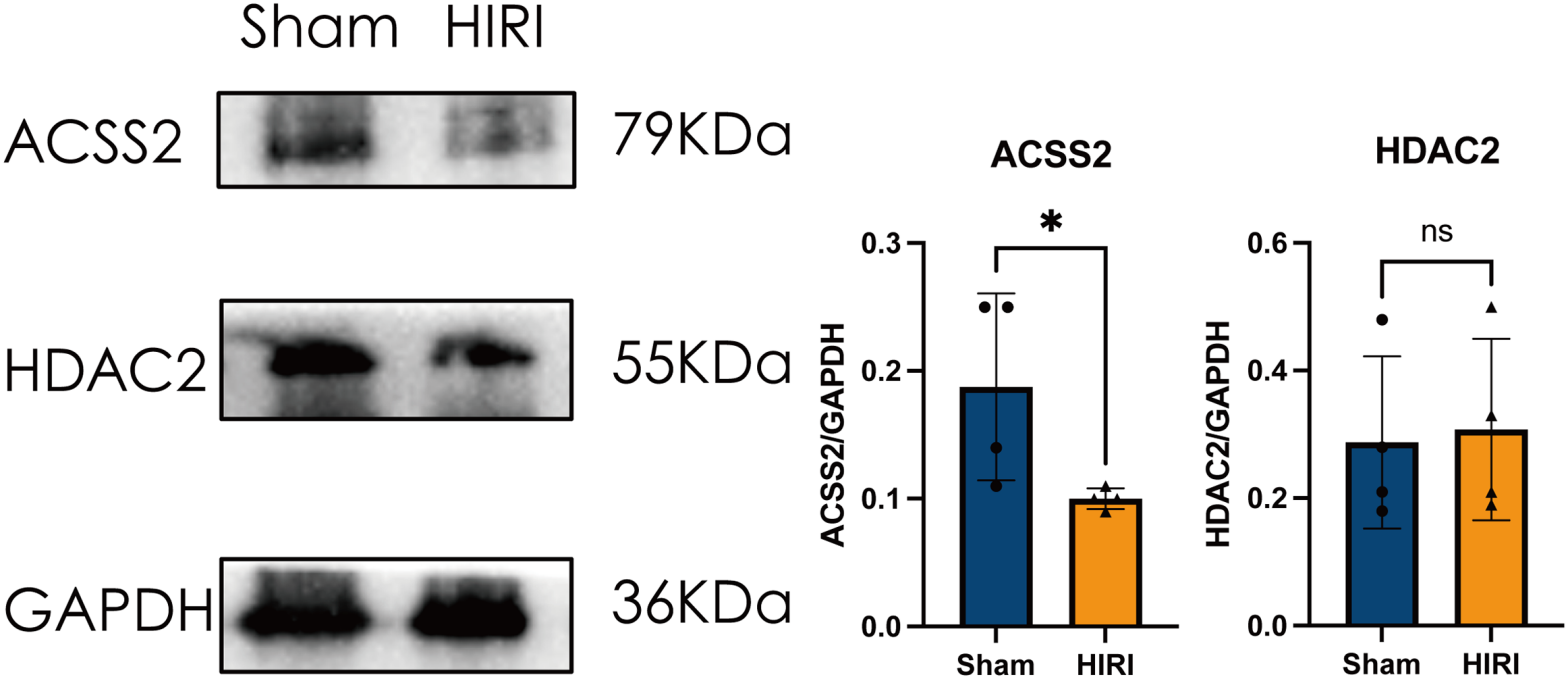
Dysregulation of HDAC2‐ACSS2 axis in the hippocampus of HIRI‐induced cognitive impairment mice. *N* = 4 for the Sham and HIRI group. Comparisons between groups were made with an unpaired *t*‐test. Data are presented as the mean ± SEM. *p < 0.05; ns, not significant.

We focused on HDAC2 and ACSS2, two key enzymes highly associated with histone acetylation/deacetylation in the hippocampus of mice. Using Western Blotting experiments, we assessed the expression of these two proteins (Figure 3). In the HIRI group, the findings indicated a notable decline in ACSS2 expression in the hippocampus of mice, whereas the expression of HDAC2 remained relatively consistent. This suggested that hepatic ischemia impairs acetate metabolism in the hippocampal region. The full unedited blots were provided in Figure S2.

### 3.3 Microbiota transplantation induced cognitive impairment and gut microbiota dysbiosis in germ‐free mice

**FIGURE 4:**
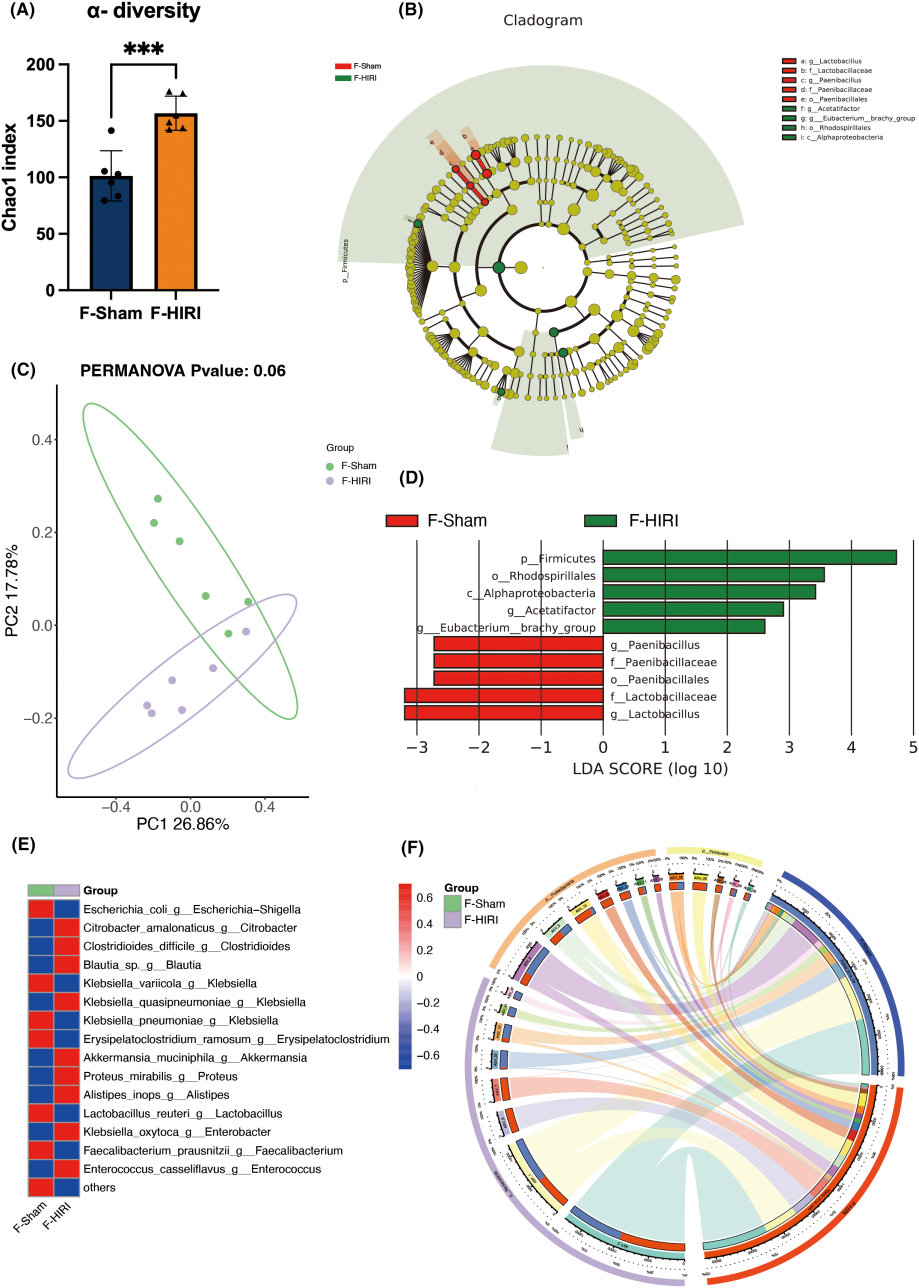
Feces microbial transplantation induced gut microbiota alternation in Pseudo germ‐free mice. (A) α‐diversity Chao1 index. (B) Principal Co‐Ordinates Analysis (PCoA). (C and D) Linear discriminant analysis Effect Size (LEfSe) analysis. (E) Top 15 different species between the two groups. (F) Circos plot. *N* = 6 for the F‐Sham and F‐HIRI group. Comparisons between groups were made with an unpaired *t*‐test. Data are presented as the mean ± SEM. ***p < 0.001 (*n* = 6 in each group).

**FIGURE 5:**
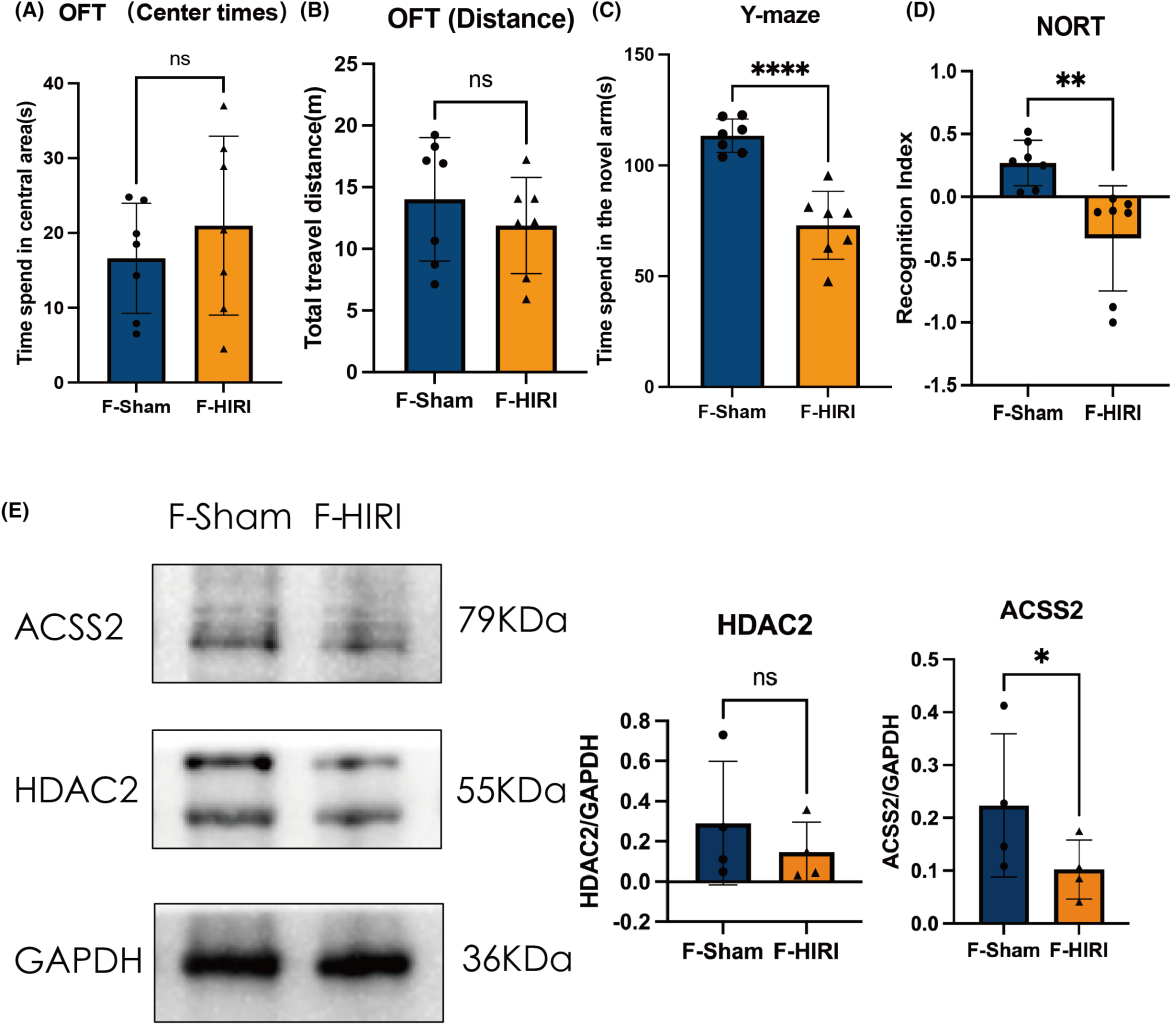
Feces microbial transplantation induced cognitive deficits and dysregulation of HDAC2‐ACSS2 axis in the hippocampus of FMT mice. (A) Time spent in the central area of OFT. (B) Total travel distance of open field test (OFT). (C) Time spent in the novel arm of the Y‐maze test. (D) Recognition index of novel object recognition test (NORT). (E) The expression level of ACSS2 and HDAC2 in the hippocampus of FMT mice. *N* = 5–7 for the F‐Sham and F‐HIRI group. Comparisons between groups were made with an unpaired *t*‐test. Data are presented as the mean ± SEM. *p < 0.05; ns, not significant.

To further validate the correlation between gut microbiota and cognitive deficits induced by HIRI, we transplanted fecal samples from the HIRI group and Sham group of mice into germ‐free mice. Similar to previous experimental results, gut microbiota dysbiosis, and cognitive dysfunction were effectively transmitted to germ‐free mice through microbiota transplantation (Figures 4 and 5). The representative images of behavior tests of F‐Sham and F‐HIRI were provided in Figure S4.

### 3.4 Microbiota transplantation induced dysregulation of the HDAC2‐ACSS2 axis and abnormal SCFA metabolism in the hippocampus of germ‐free mice

**FIGURE 6:**
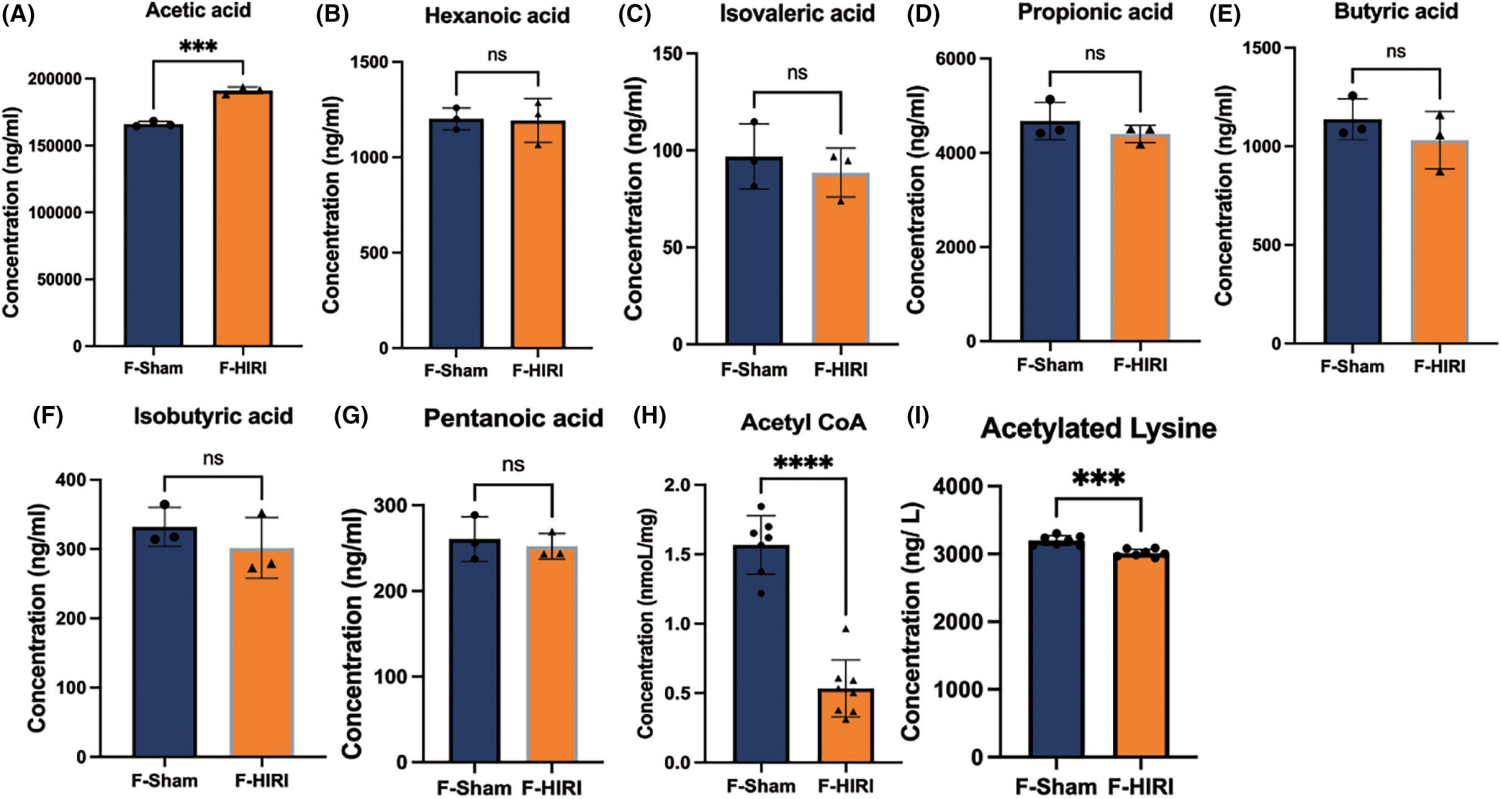
The levels of short‐chain fatty acids (SCFAs), acetyl CoA and Acetylated Lysine in the hippocampus in FMT mice. (A–G) Comparison of the SCFA levels in the hippocampus between F‐Sham and F‐HIRI. (H) Comparison of the acetyl CoA levels in the hippocampus between F‐Sham and F‐HIRI. (I) Comparison of the Acetylated Lysine levels in the hippocampus between F‐Sham and F‐HIRI. *N* = 3–6 for the F‐Sham and F‐HIRI group. Comparisons between groups were made with an unpaired *t*‐test. Data are presented as the mean ± SEM. ***p < 0.001, ****p < 0.0001; ns, not significant.

Surprisingly, the phenomenon of HDAC2‐ACSS2 axis dysregulation was also successfully transmitted to germ‐free mice through microbiota transplantation (Figure 5). The full unedited blots were exhibited in Figure S2. In order to comprehend the metabolic process of SCFAs in the hippocampus of FMT mice, we additionally assessed the quantities of different typical SCFAs and acetyl‐CoA in the hippocampus of both sets (Figure 6A–G). The results suggested that mice receiving microbiota transplantation from the HIRI group had significantly higher acetate levels in the hippocampus compared to mice receiving transplantation from the Sham group. Simultaneously, there was a significant decrease in the acetyl‐CoA level and acetylated lysine level in the hippocampus of mice in the F‐HIRI group, indicating potential disruptions in the utilization of acetate, synthesis of acetyl‐CoA, and histone acetylation in the hippocampus of these mice (Figure 6H,I).

## 4 DISCUSSION

**FIGURE 7:**
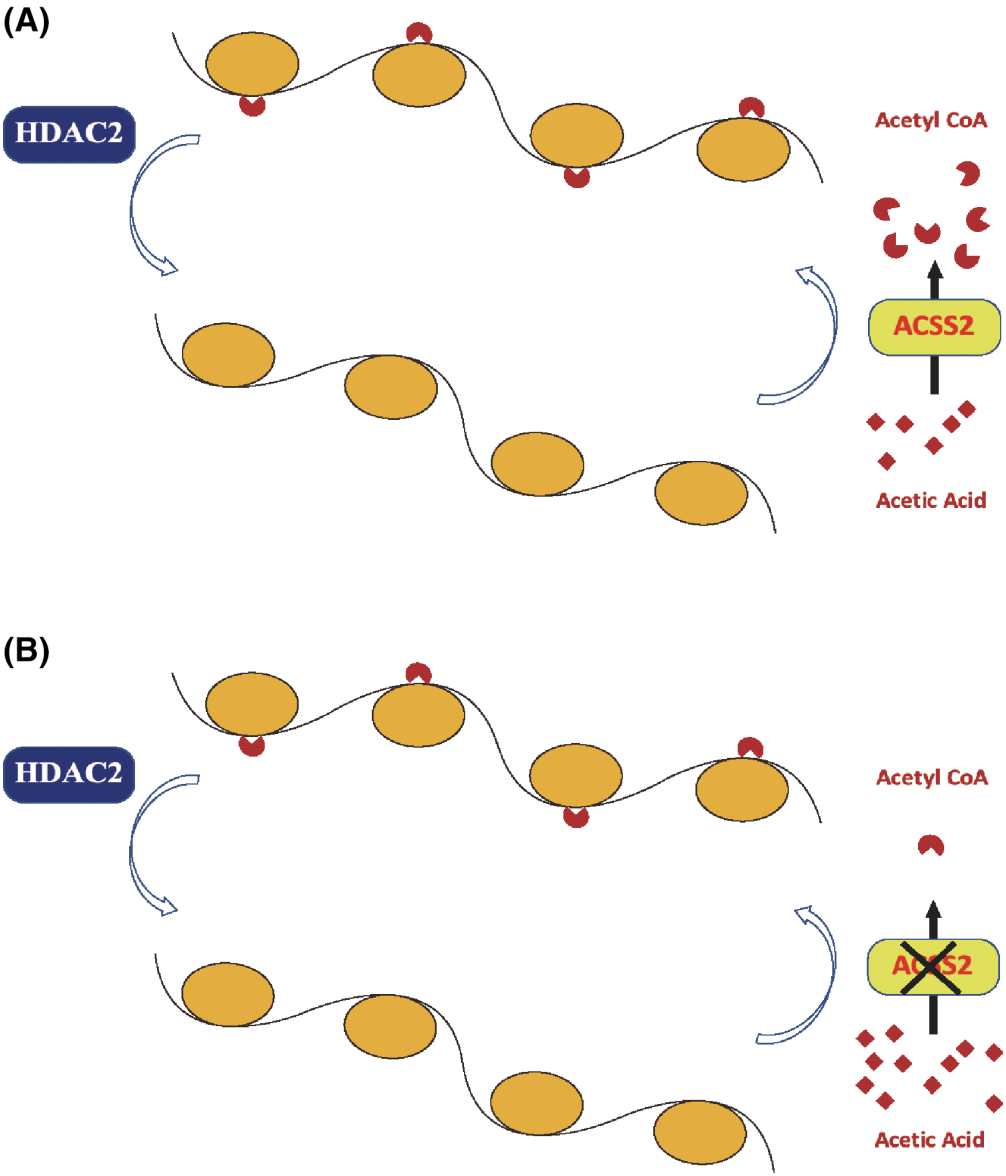
A hypothetical mechanism for disturbances in acetate utilization, acetyl‐CoA synthesis, and histone acetylation in the hippocampus of HIRI‐induced cognitive impairment mice. (A) Normal mice. (B) HIRI‐induced cognitive dysfunction mice and FMT mice.

The present study utilized multiple techniques, including gut microbiota analysis, behavioral tests, and LC/MS SCFA profiling to demonstrate the existence of HIRI‐induced cognitive deficit in mice subjected to HIRI during nighttime. Additionally, western blotting (WB) analysis revealed a significant dysregulation in ACSS2 expression in the hippocampus of rodents with HIRI, indicating impaired ACSS2 function in this region. Furthermore, the cognitive impairment phenotype and the decreased ACSS2 expression could be transmitted to germ‐free mice through FMT. Further examinations of levels of SCFA in the hippocampus of transplanted germ‐free mice highlighted a significant reduction in acetate levels in mice receiving cognitive impairment‐associated gut microbiota, accompanied by a marked decrease in acetyl‐coenzyme A (acetyl‐CoA) levels. These findings suggest impaired acetate metabolism in the hippocampus of cognitive impairment mice that can lead to compromised histone acetylation (Figure 7).

Furthermore, 16S rRNA analysis revealed significant differences in several SCFA metabolism and inflammatory response‐related gut microbial taxa, such as *Anaerofustis*, *Blautia*, *Akkermansia muciniphila*, and *Alistipes*, between the HIRI and Sham groups, as well as the F‐HIRI and F‐Sham groups. These changes may be correlated with cognitive impairment and hippocampal acetate metabolic dysregulation, possibly contributing to the development of cognitive impairment following fecal microbial transplantation.^37^, ^38^, ^39^, ^40^, ^41^, ^42^, ^43^ However, this study did not elucidate the specific underlying mechanisms, and further investigations are warranted to address this issue.

Nevertheless, the current study has some limitations. Firstly, the precise reasons behind the gut microbiota‐induced HDAC2‐ACSS2 axis imbalance are not extensively explored. Previous studies have implicated alterations in hippocampal SCFA levels (e.g., significantly reduced acetate levels in the experimental group compared to the control group) in cognitive impairment models induced by various factors, which is related to the gut microbial SCFA metabolism.^44^, ^45^, ^46^ The results of this study differ somewhat from the aforementioned findings, suggesting that the impact of gut microbial metabolites, such as SCFAs, on cognitive function may vary under different conditions. Therefore, further in‐depth research is needed. Secondly, due to experimental constraints, we were unable to confirm our conclusions by directly measuring histone acetylation levels.

HIRI, as one of the most prevalent complications in liver surgery, has been a focus of research in the academic community. The present understanding of the etiology of HIRI mainly attributes it to the damage caused by oxygen‐free radicals upon blood reperfusion, resulting in cell death due to calcium overload, and immune system‐related actions. Similarly, these damages can exacerbate inflammation in the central nervous system and promote the development of cognitive impairment. The present study provides a novel research perspective on the development of cognitive impairment caused by HIRI, offering a theoretical basis for subsequent clinical treatments.

## CONFLICT OF INTEREST STATEMENT

The researchers affirm that the study was carried out without any business or monetary affiliations that could be interpreted as a possible clash of interests.

## Supporting information


Figure S1.

Figure S2.

Figure S3.

Figure S4.

Data S1.

## Data Availability

The data that support the findings of this study are available from the corresponding author upon reasonable request.
