# Supplementary figures and images for: Gut microbiota regulates hepatic ischemia–reperfusion injury‐induced cognitive dysfunction via the HDAC2‐ACSS2 axis in mice
Source: CNS Neurosci Ther. 2024 Feb 9;30(2):e14610. doi: 10.1111/cns.14610 (PMC10853894; doi:10.1111/cns.14610)

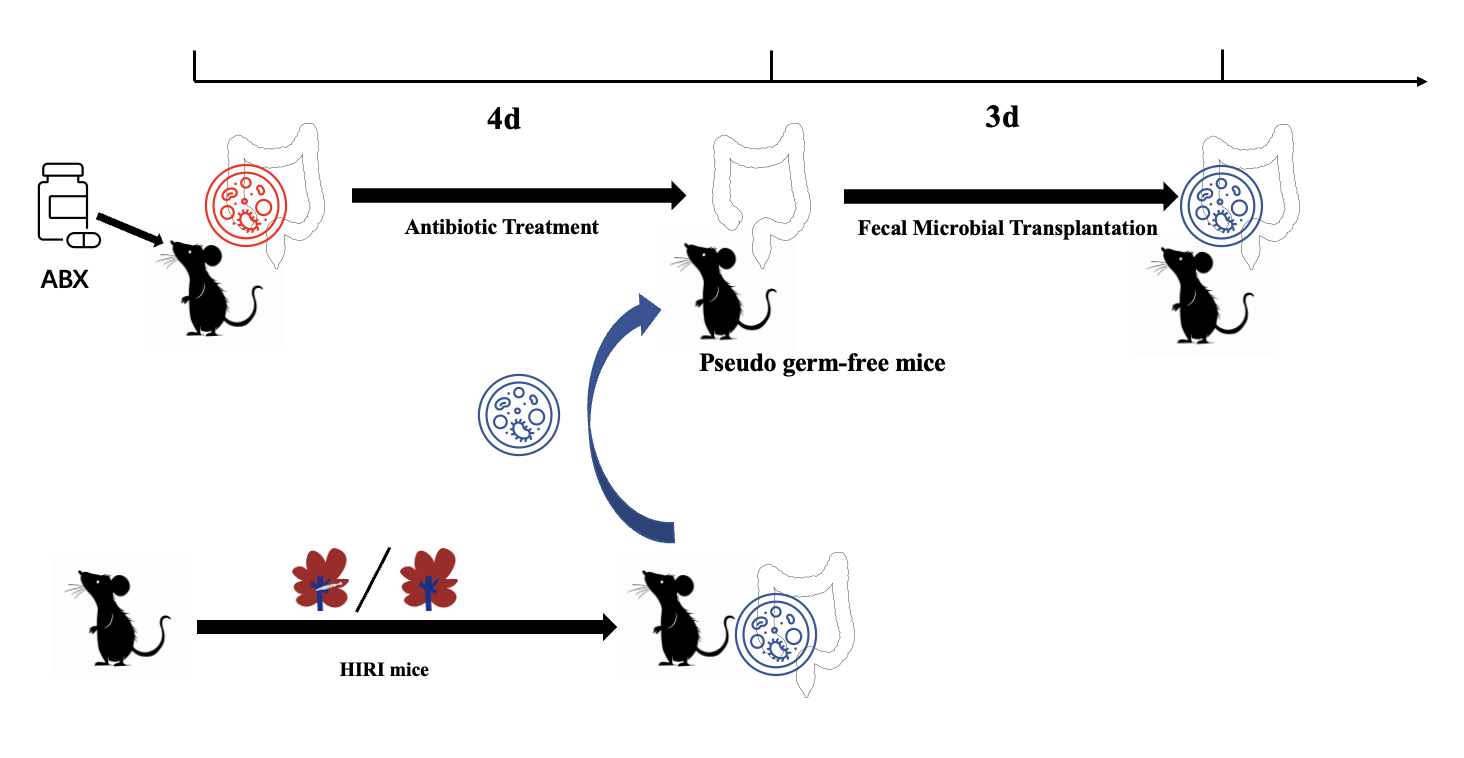

Supplement: Supplementary file 1 — Figure S1. [file CNS-30-e14610-s002.png]

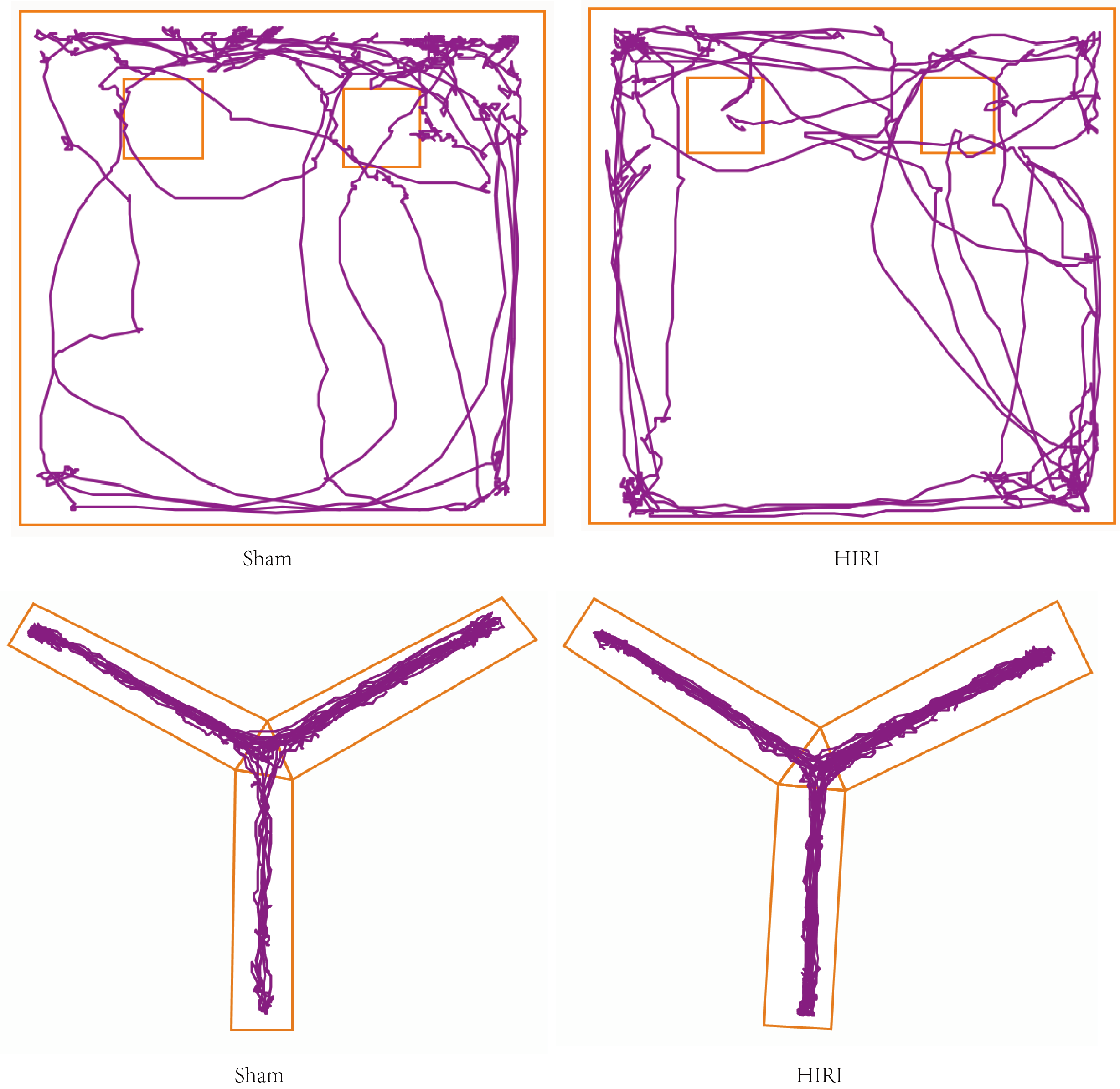

Supplement: Supplementary file 3 — Figure S3. [file CNS-30-e14610-s005.png]

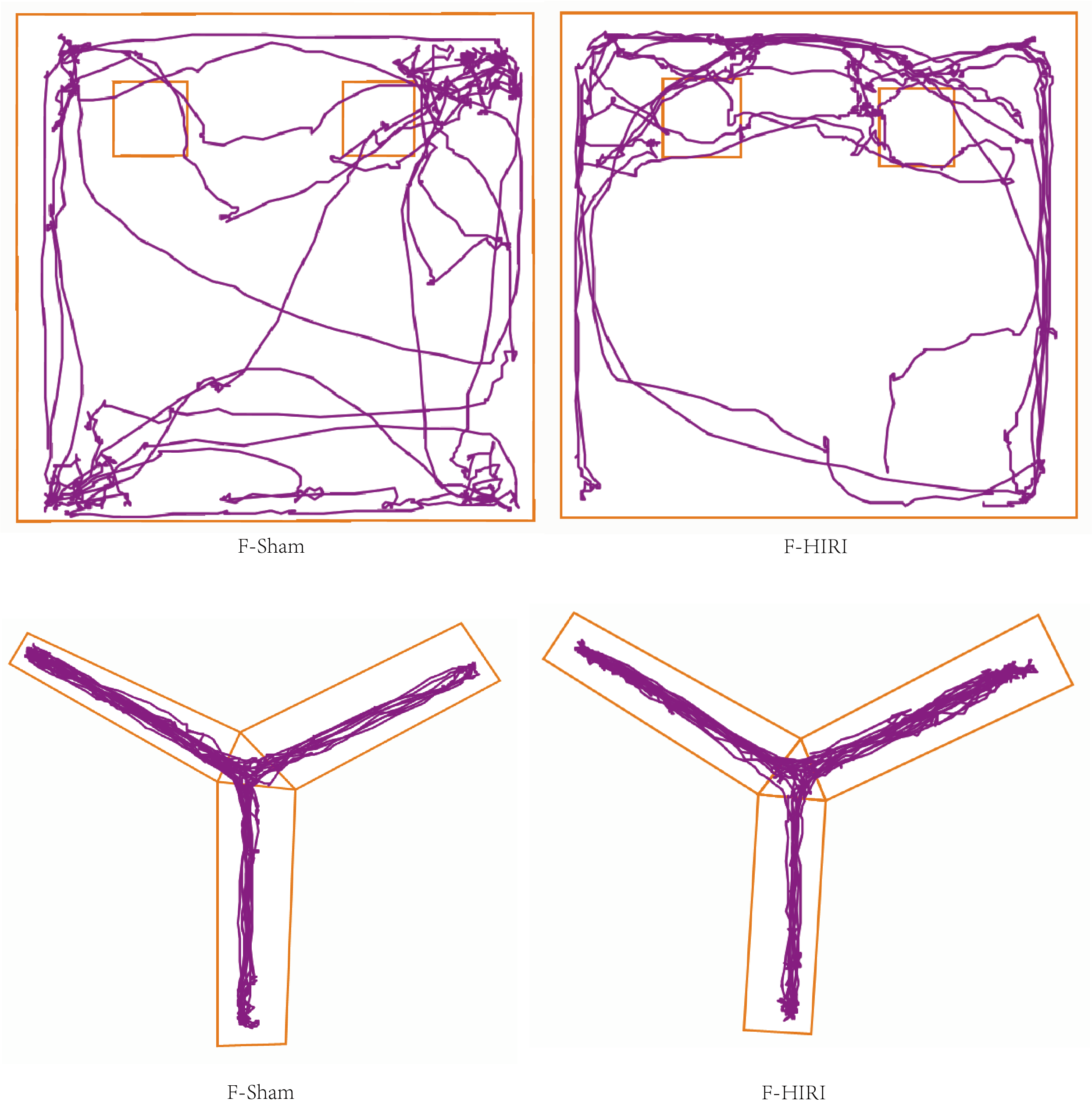

Supplement: Supplementary file 4 — Figure S4. [file CNS-30-e14610-s003.png]
