# Supplementary material for: Gut microbiota regulates hepatic ischemia–reperfusion injury‐induced cognitive dysfunction via the HDAC2‐ACSS2 axis in mice
Source: CNS Neurosci Ther. 2024 Feb 9;30(2):e14610. doi: 10.1111/cns.14610 (PMC10853894; doi:10.1111/cns.14610)
Supplement: Supplementary file 2 — Figure S2. [file CNS-30-e14610-s004.pdf]

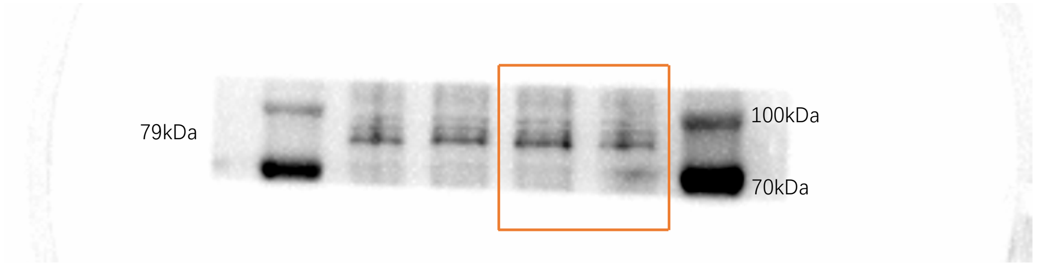

Full unedited blot for Figure 5（ACSS2）

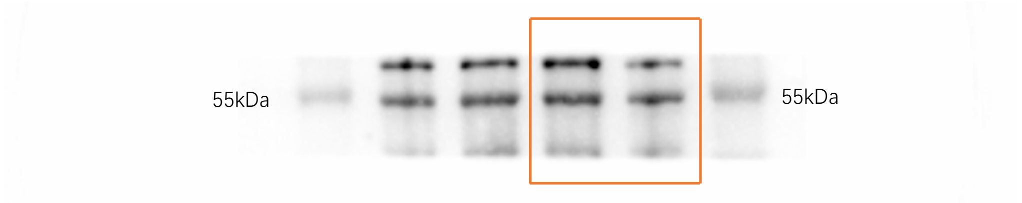

Full unedited blot for Figure 5（HDAC2）

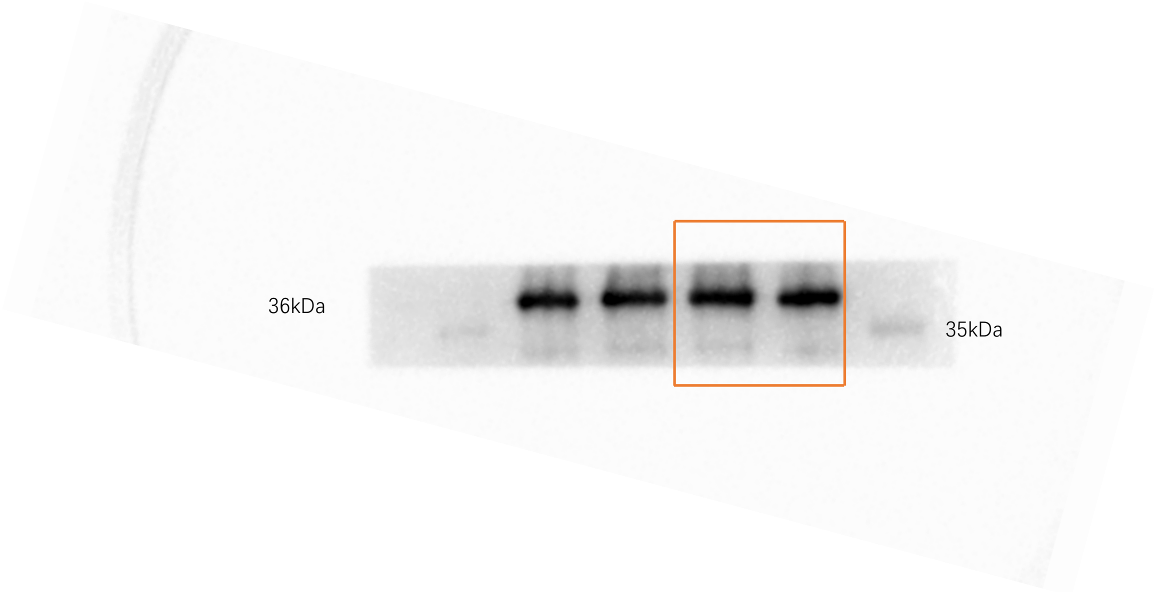

Full unedited blot for Figure 5（GAPDH）

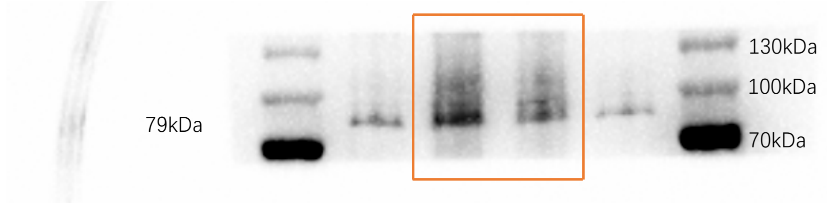

Full unedited blot for Figure 1（ACSS2）

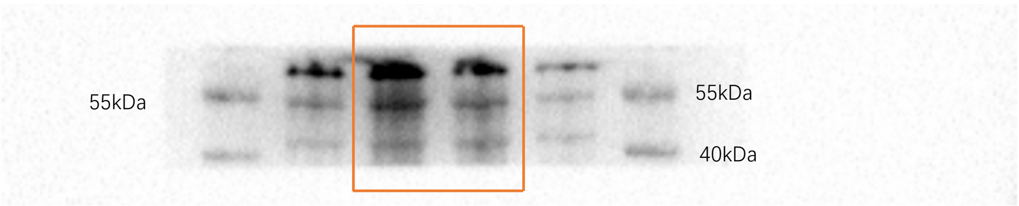

Full unedited blot for Figure 1（HDAC2）

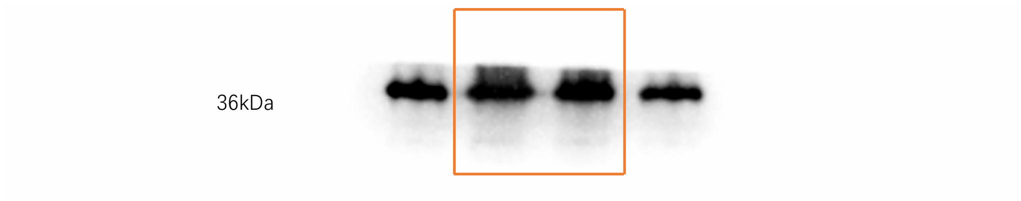

Full unedited blot for Figure 1（GAPDH）
