# Supplementary material for: Gut microbiota regulates hepatic ischemia–reperfusion injury‐induced cognitive dysfunction via the HDAC2‐ACSS2 axis in mice
Source: CNS Neurosci Ther. 2024 Feb 9;30(2):e14610. doi: 10.1111/cns.14610 (PMC10853894; doi:10.1111/cns.14610)
Supplement: Supplementary file 5 — Data S1. [file CNS-30-e14610-s001.docx]

Supplementary Figure 1 Fecal Microbiome Transplantation Procedure.

Supplementary Figure 2 Full unedited blot of Western Blot.

Supplementary Figure 3 The representative images of behavior tests of Sham and HIRI groups.

Supplementary Figure 4 The representative images of behavior tests of F-Sham and F-HIRI groups.
